# Cardiac Device-Related Infective Endocarditis Presenting With Suspected Embolic Stroke in a Patient With a Legacy Implantable Cardioverter-Defibrillator: A Case Report

**DOI:** 10.7759/cureus.113354

**Published:** 2026-07-25

**Authors:** Samuel Schwartz, Abraham E Libman, Ahmad Naimi, Mayesha Ahmed, Roxana Lazarescu

**Affiliations:** 1 Internal Medicine, Touro College of Osteopathic Medicine, New York, USA; 2 Internal Medicine, Wyckoff Heights Medical Center, New York, USA

**Keywords:** cardiac implantable electronic device, device-related endocarditis, embolic stroke, group b streptococcus, icd lead infection, infective endocarditis, lead vegetation, legacy icd, streptococcus agalactiae, transesophageal echocardiography

## Abstract

Cardiac device-related infective endocarditis can evade early recognition when embolic neurologic symptoms obscure the underlying cardiac source. We report the case of an 87-year-old man with a cardiac resynchronization therapy defibrillator (CRT-D) that was not labeled as MRI-conditional. He presented after an unwitnessed fall with acute dysarthria, facial asymmetry, and asymmetric extremity weakness, with a National Institutes of Health Stroke Scale score of 10 during the acute stroke evaluation. Initial cardiovascular examination revealed no murmur, and noncontrast CT of the head showed no acute intracranial abnormality. Diffusion-weighted MRI could not be obtained at the presenting institution because of his non-MRI-conditional cardiac hardware. Over the subsequent hospital course, his condition progressed to multiorgan failure, marked by worsening baseline thrombocytopenia, acute renal dysfunction, and hyperbilirubinemia. Admission blood cultures grew *Streptococcus agalactiae*, prompting transesophageal echocardiography (TEE), which demonstrated a mobile vegetation attached to the CRT-D lead and confirmed lead-associated infective endocarditis. This case illustrates how a non-MRI-conditional cardiac implantable electronic device (CIED) can limit neuroimaging in patients with suspected cerebral ischemia and systemic infection. Clinicians should consider early TEE when focal neurologic deficits are accompanied by fever, bacteremia, or systemic toxicity in a patient with a CIED, even when the cardiovascular examination and initial CT neuroimaging are unrevealing.

## Introduction

The growing use of cardiac implantable electronic devices (CIEDs) in older adults has made hardware-associated infections more difficult to recognize. CIED-related infective endocarditis carries substantial morbidity and mortality, with reported in-hospital and one-year mortality rates of 14.7% and 23.2%, respectively [1]. Although fever and bacteremia are common, CIED-related infective endocarditis may differ from classic native valve endocarditis because lead-associated infections may not produce a new auscultatory murmur that prompts early cardiac evaluation [2].

Infective endocarditis may present with neurologic complications before the cardiac source is recognized. These complications include ischemic stroke from septic embolization, intracerebral hemorrhage, infectious intracranial aneurysm, and cerebral abscess. Stroke occurs in approximately 20-40% of cases and may precede other findings [3,4]. The initial clinical picture may therefore suggest a primary cerebrovascular event while the underlying endovascular source remains occult.

We report the case of a patient whose acute neurologic presentation prompted a stroke evaluation, but whose cardiac resynchronization therapy defibrillator (CRT-D) was not labeled as MRI-conditional, limiting access to diffusion-weighted MRI (DWI-MRI) at the presenting institution. The CRT-D lead was subsequently identified as the infectious source. This case demonstrates a diagnostic blind spot in which focal neurologic deficits may direct attention toward primary stroke evaluation while non-MRI-conditional hardware limits the assessment of small or early ischemic lesions. When these deficits occur alongside systemic signs of infection, unrevealing head CT should not provide false reassurance. This combination should lower the threshold for early transesophageal echocardiography (TEE). In this report, the term “legacy” in the title refers to a non-MRI-conditional device system; the device is otherwise referred to as a CRT-D.

## Case presentation

An 87-year-old man presented to the ED after an unwitnessed fall with an estimated time on the ground of seven hours. Family members reported that fever and progressive confusion began approximately 48 hours before admission. He had remained ambulatory until the fall, and focal neurologic deficits were first documented during the ED evaluation. His medical history included heart failure with a previously documented ejection fraction of 39%, atrial fibrillation status post left atrial appendage occlusion (LAAO) device implantation in 2016, and a non-MRI-conditional CRT-D. Information regarding the underlying etiology of his heart failure, the original indication for CRT-D implantation, longitudinal cardiology or device clinic follow-up, prior device-related complications or interventions, and the complete preadmission medication list was unavailable.

On arrival, cardiovascular examination revealed normal first and second heart sounds (S1 and S2) without murmurs, rubs, or gallops. Neurologic examination showed dysarthria, facial asymmetry, and asymmetric extremity weakness, with left lower-extremity strength of 3/5 and right upper-extremity strength of 2/5. His National Institutes of Health Stroke Scale (NIHSS) score was 10 during the acute stroke evaluation [5]. Noncontrast head CT showed no acute hemorrhage or established infarct. Additional trauma imaging was unremarkable. An acute cerebrovascular event remained the initial working diagnosis. However, DWI-MRI could not be obtained at the presenting institution because of the patient’s non-MRI-conditional CRT-D, limiting radiographic evaluation for small or early embolic ischemia. His neurologic deficits subsequently resolved, and he returned to baseline function.

Initial laboratory testing showed thrombocytopenia below his reported baseline platelet count of approximately 90-100 × 10³/µL, along with hyperbilirubinemia, acute renal dysfunction, and elevated cardiac biomarkers. During the subsequent hospital course, worsening uremia and sepsis-associated hepatic injury reflected evolving multiorgan dysfunction. Key admission and follow-up laboratory values are summarized in Table 1.

**Table 1:** Selected laboratory trends during multiorgan dysfunction and clinical recovery. Values in the hospital days 4-9 column represent interval values unless a specific hospital day is indicated in parentheses.

Lab value (reference range/expected result)	Hospital days 1-4 (admission)	Hospital days 4-9 (nadir)	Hospital day 17 (transfer)
Hematology			
White blood cell count (4.5-11.0 K/µL)	6.1	13.3	5.4
Hemoglobin (13.0-17.0 g/dL)	13.3	12.5	10.4
Platelet count (130-400 K/µL)	40	31 (day 4)	147
Renal and metabolic markers			
Creatinine (0.55-1.30 mg/dL)	1.96	2.17	0.97
Blood urea nitrogen (7-18 mg/dL)	48	104	16
Lactic acid (0.4-2.0 mmol/L)	2.4	2.7	1.6
Hepatobiliary markers			
Total bilirubin (0.2-1.0 mg/dL)	1.6	8.2 (day 9)	3.4
Direct bilirubin (0.10-0.20 mg/dL)	1.25 (day 3)	5.11	1.38
Alkaline phosphatase (45-117 U/L)	140	272	126
Aspartate aminotransferase (15-37 U/L)	34	226	48
Alanine aminotransferase (12-78 U/L)	20	-	19
Cardiac biomarkers			
High-sensitivity troponin I (3.0-58.9 ng/L)	115.9	113.2	-
B-type natriuretic peptide (0-100 pg/mL)	531.7	-	-
Microbiology			
Blood culture (expected result: no growth)	Pending	*Streptococcus agalactiae*	No growth

**Figure 1:**
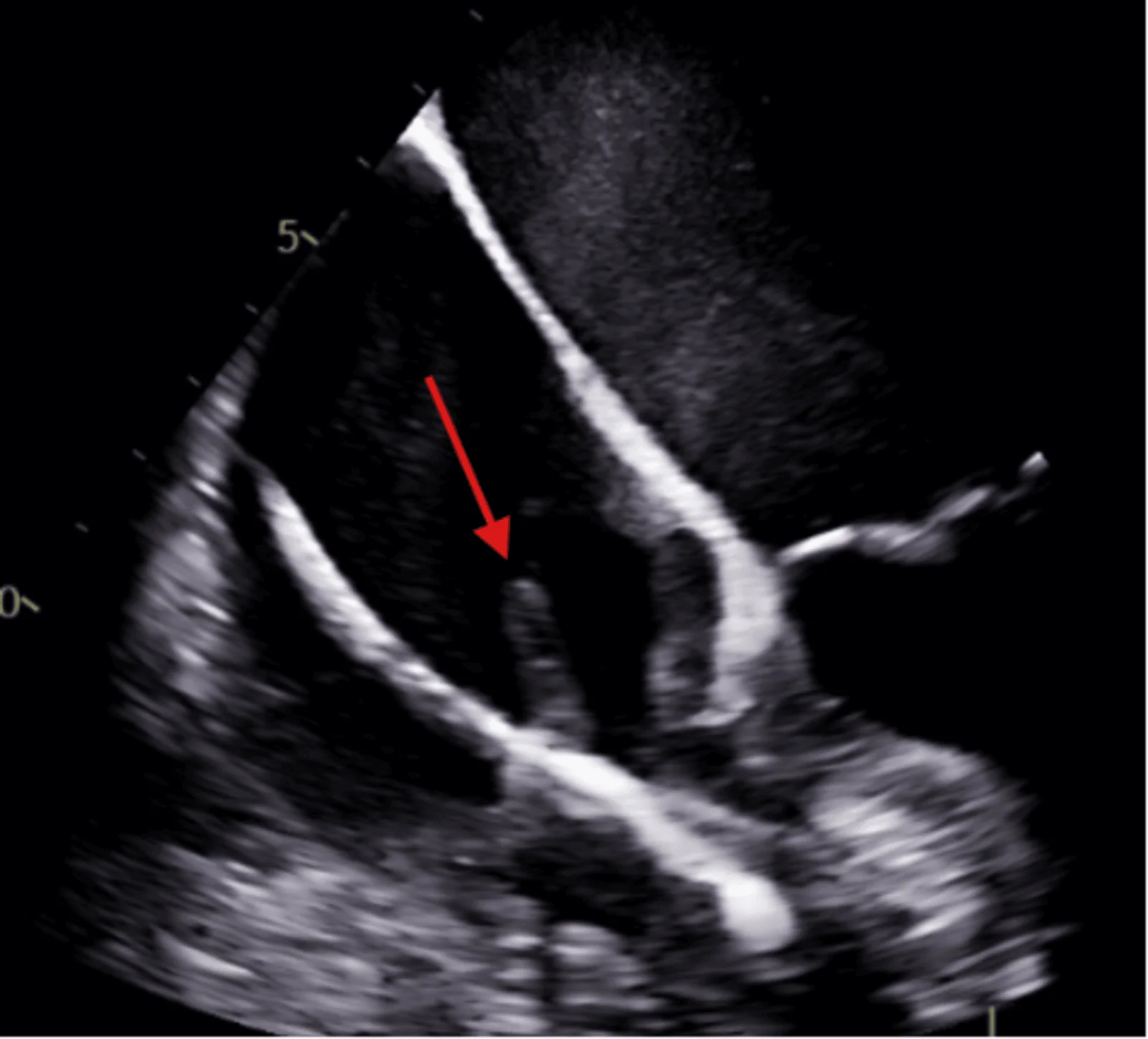
CRT-D lead vegetation. TEE image showing a mobile echodensity attached to the CRT-D lead (arrow), consistent with lead-associated vegetation in the setting of *Streptococcus agalactiae *bacteremia. CRT-D, cardiac resynchronization therapy defibrillator; TEE, transesophageal echocardiography

**Figure 2:**
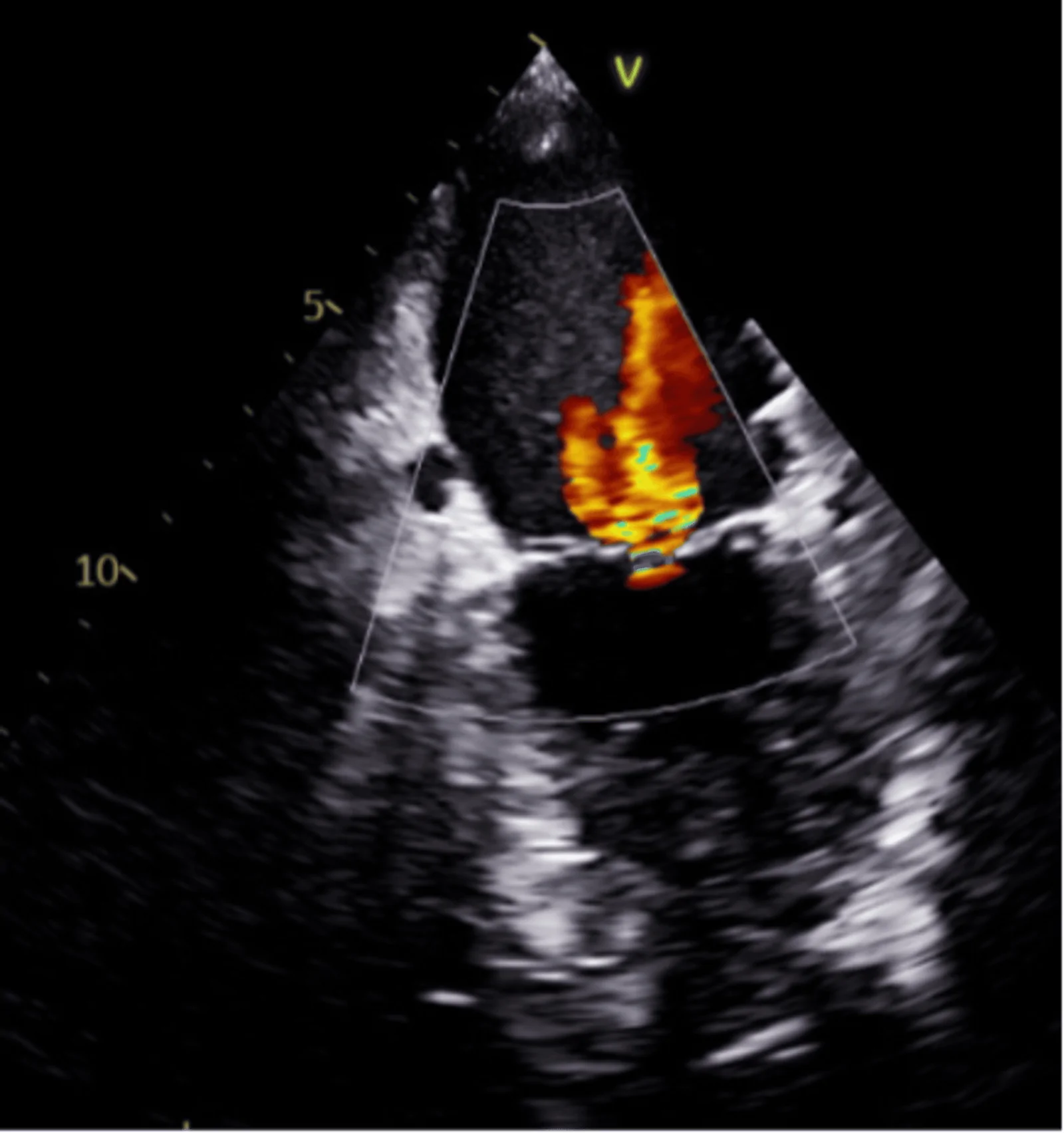
Mitral regurgitation. Color Doppler TEE image showing mitral regurgitation, demonstrated by a regurgitant jet extending into the left atrium. TEE, transesophageal echocardiography

**Figure 3:**
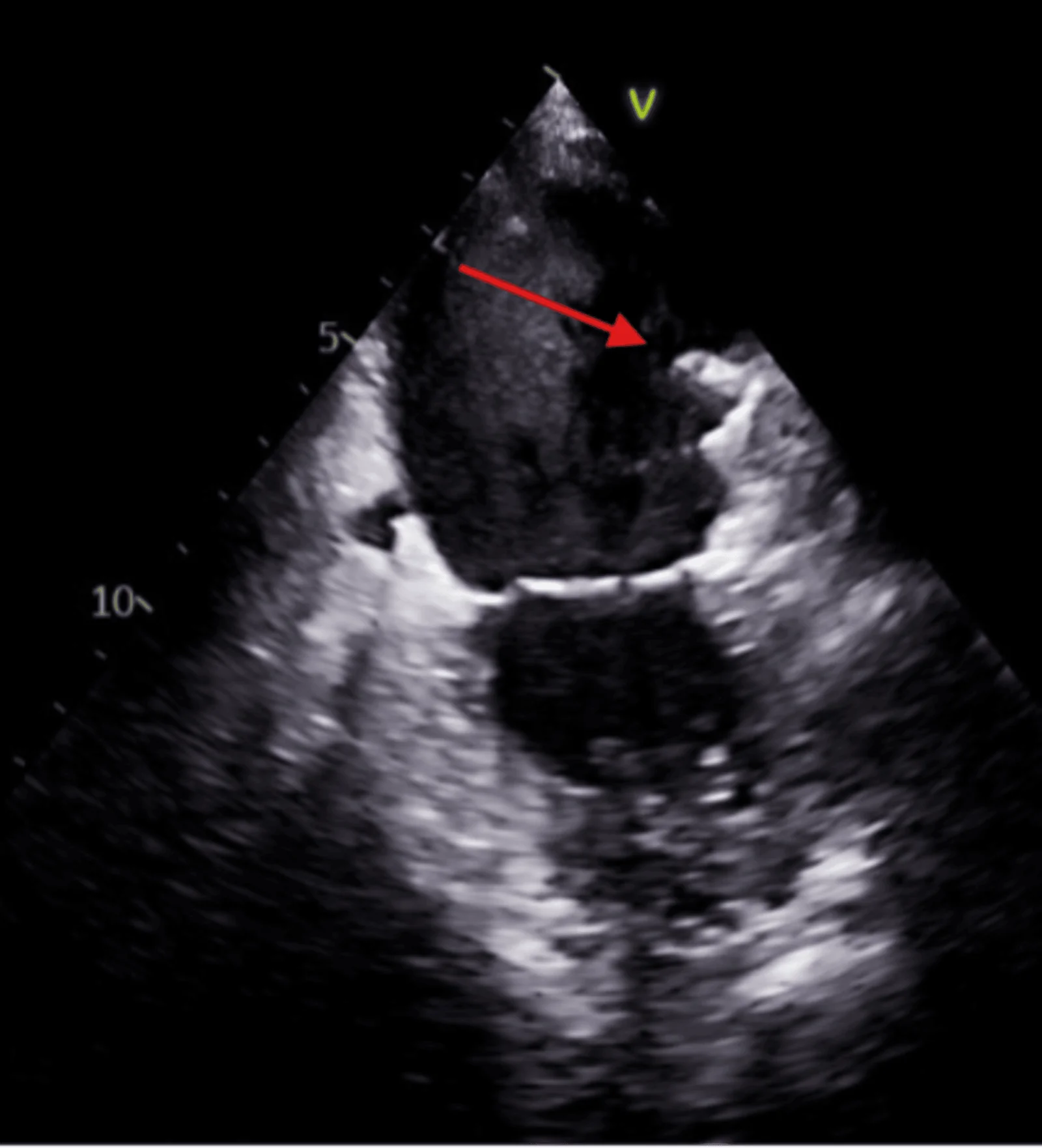
LAAO device. TEE image showing the patient’s LAAO device (arrow). LAAO, left atrial appendage occlusion; TEE, transesophageal echocardiography

On hospital day 4, blood cultures grew *Streptococcus agalactiae*, also known as group B *Streptococcus *(GBS). The records available for this report did not specify the number of positive blood culture sets or bottles and did not permit confirmation of whether a formal infectious diseases consultation occurred before transfer. Transthoracic echocardiography (TTE), obtained on the same day, showed an ejection fraction of 40-45%, with mild mitral and tricuspid regurgitation. Given persistent concern for an occult endovascular source in the setting of GBS bacteremia and indwelling cardiac hardware, TEE was performed on hospital day 8 and demonstrated a mobile echodensity attached directly to the CRT-D lead (Figure 1). Color Doppler imaging showed mild mitral regurgitation across thickened leaflets, and tricuspid regurgitation was also noted (Figure 2). The LAAO device was visualized on TEE, with no device-associated abnormality described in the report (Figure 3). These findings confirmed lead-associated infective endocarditis.

The patient received empiric piperacillin-tazobactam on admission. After organism identification and susceptibility testing, antimicrobial therapy was narrowed to penicillin G and, on hospital day 11, transitioned to intravenous ceftriaxone 2 g every 12 hours. The rationale for this change was not documented in the records available to the authors. He stabilized clinically, and his platelet count recovered toward baseline. He was subsequently transferred to a tertiary care facility for lead extraction. Records from the receiving institution were unavailable; therefore, extraction findings, total antimicrobial duration, and post-transfer clinical outcomes could not be reported.

## Discussion

This case is not presented as evidence that neurologic complications of infective endocarditis or the absence of a murmur in device-related infection are novel. Its educational value lies in the need to pursue neurologic and endovascular evaluations in parallel when focal deficits occur with fever, bacteremia, or multiorgan dysfunction in a patient with a CIED. The NIHSS score of 10 appropriately prompted urgent stroke evaluation, while the concurrent systemic findings warranted evaluation for an endovascular source. The non-MRI-conditional CRT-D limited the acute neuroimaging workup at the presenting institution.

MRI is more sensitive than CT for detecting cerebral lesions in infective endocarditis [6]. In patients with symptomatic cerebral complications, MRI detected lesions with 100% sensitivity compared with 81% for CT [7]. MRI findings may also influence management, as cerebral embolic lesions satisfy a minor diagnostic criterion for infective endocarditis and can alter surgical timing [6,8].

In this patient, head CT ruled out acute hemorrhage, and his neurologic deficits later resolved with return to baseline function. However, because DWI-MRI could not be obtained, a transient ischemic event or small embolic infarct could not be radiographically confirmed. Accordingly, the neurologic event remained suspected rather than radiographically confirmed, and a direct causal relationship between the lead infection and the transient deficits could not be established.

Non-MRI-conditional pacemakers and defibrillators carry established risks during MRI, including lead tip heating and device malfunction. Nazarian et al. demonstrated that MRI can be performed safely in patients with non-MRI-conditional devices under strict institutional protocols [9]. However, widespread implementation remains limited. The 2017 Heart Rhythm Society Expert Consensus Statement requires formal protocols, designated CIED physicians, continuous monitoring, and immediately available programming equipment for patients with non-MRI-conditional devices [10]. In many nontertiary settings, these requirements render acute MRI largely inaccessible in standard practice.

In the setting of bacteremia and indwelling cardiac hardware, the diagnostic evaluation should extend beyond neuroimaging to identify a potential endovascular source. TEE remains the preferred modality for evaluating suspected cardiac device hardware infection because it provides superior spatial resolution, reduces artifact from prosthetic material, and better visualizes lead-associated vegetations than TTE [11,12]. In this case, TEE established the diagnosis by identifying the vegetation directly on the CRT-D lead. Earlier echocardiography might have shortened the time to diagnosis and is acknowledged as a limitation rather than proposed as an ideal diagnostic sequence.

Beyond imaging limitations, physical examination findings can also mislead clinicians. Murmurs are a hallmark of native valve endocarditis, but lead-associated infections often spare the valve leaflets. Vegetations confined to device leads may not produce new auscultatory findings [2]. As this case illustrates, a benign cardiovascular examination may provide false reassurance, especially when intracardiac hardware and unexplained systemic symptoms are present.

The patient’s laboratory abnormalities reflected severe systemic illness (Table 1). Thrombocytopenia and hyperbilirubinemia independently predict poor clinical outcomes in infective endocarditis [13,14]. Elevated high-sensitivity troponin I is common in infective endocarditis and has been identified as one of the strongest independent predictors of in-hospital mortality among multiple biomarkers [15]. Together, these abnormalities, GBS bacteremia, and indwelling cardiac hardware heightened concern for device-related infective endocarditis and lowered the threshold for TEE, consistent with guideline recommendations for suspected endocarditis in patients with intracardiac device leads [16].

The identification of GBS was notable because this organism is classically associated with pregnancy and neonatal infection, yet invasive GBS disease is increasingly recognized among older nonpregnant adults with chronic comorbidities [17]. GBS endocarditis is uncommon but often clinically aggressive, with reported complications including major emboli, heart failure, and shock [18]. In this case, the microbiologic findings support a clinically plausible association among invasive GBS infection, lead-associated endocarditis, suspected embolic neurologic involvement, and rapid systemic deterioration.

Limitations

This report is limited by incomplete retrospective records. Complete details regarding the underlying heart failure etiology, the original indication for CRT-D implantation and longitudinal device follow-up, outpatient medications, the number of positive blood culture sets or bottles, infectious diseases consultation, the rationale for the antibiotic change, and the post-transfer course were unavailable. The absence of brain MRI also prevented radiographic characterization of the neurologic event.

## Conclusions

Cardiac device-related infective endocarditis may be identified during the evaluation of transient focal neurologic deficits, although the neurologic event should not be presumed to represent septic embolism when confirmatory imaging is unavailable. When focal deficits occur together with fever, bacteremia, or multiorgan dysfunction in a patient with a CIED, neurologic and endovascular evaluations should proceed in parallel. In this case, TEE established the diagnosis of CRT-D lead-associated infective endocarditis after the initial cardiovascular examination and head CT were unrevealing.
